# APOE Genotype Effects on Intrinsic Brain Network Connectivity in Patients with Amnestic Mild Cognitive Impairment

**DOI:** 10.1038/s41598-017-00432-0

**Published:** 2017-03-24

**Authors:** Zan Wang, Zhengjia Dai, Hao Shu, Xuhong Liao, Chunxian Yue, Duan Liu, Qihao Guo, Yong He, Zhijun Zhang

**Affiliations:** 1 Department of Neurology, Affiliated ZhongDa Hospital, School of Medicine, Southeast University, Nanjing, Jiangsu 210009 China; 2 Department of Psychology, Sun Yat-sen University, Guangzhou, 510006 China; 3 State Key Laboratory of Cognitive Neuroscience and Learning & IDG/McGovern Institute for Brain Research, Beijing Normal University, Beijing, 100875 China; 4 Department of Neurology, Huashan Hospital, Fudan University, Shanghai, 200040 China

## Abstract

Whether and how the apolipoprotein E (APOE) ε4 genotype specifically modulates brain network connectivity in patients with amnestic mild cognitive impairment (aMCI) remain largely unknown. Here, we employed resting-state (‘task-free’) functional MRI and network centrality approaches to investigate local (degree centrality, DC) and global (eigenvector centrality, EC) functional integrity in the whole-brain connectome in 156 older adults, including 66 aMCI patients (27 ε4-carriers and 39 non-carriers) and 90 healthy controls (45 ε4-carriers and 45 non-carriers). We observed diagnosis-by-genotype interactions on DC in the left superior/middle frontal gyrus, right middle temporal gyrus and cerebellum, with higher values in the ε4-carriers than non-carriers in the aMCI group. We further observed diagnosis-by-genotype interactions on EC, with higher values in the right middle temporal gyrus but lower values in the medial parts of default-mode network in the ε4-carriers than non-carriers in the aMCI group. Notably, these genotype differences in DC or EC were absent in the control group. Finally, the network connectivity DC values were negatively correlated with cognitive performance in the aMCI ε4-carriers. Our findings suggest that the APOE genotype selectively modulates the functional integration of brain networks in patients with aMCI, thus providing important insight into the gene-connectome interaction in this disease.

## Introduction

Alzheimer’s disease (AD) is a neurodegenerative disease characterized by the progressive impairment of cognitive and memory functions. Amnestic mild cognitive impairment (aMCI) represents a transition state between normal aging and AD, and it has a high risk of developing clinically probable AD^1^. Thus, aMCI resembles the pre-dementia stages of AD in the majority of cases and provides an important model to study the mechanisms of this disease. Nonetheless, the prognosis of aMCI patients is highly variable; some patients retain stable or even revert to a normal state, while others progress to dementia due to the interplay among genetic, physiological and environmental factors^2, 3^.

The apolipoprotein E (APOE) ε4 allele is the most common genetic variant associated with AD^4^ especially in asian population^5^. The neuropathological effects of APOE ε4-allele are myriad and include the following^6^: (1) impaired neurite outgrowth; (2) cytoskeletal disruption and hyperphosphorylation of tau; (3) mitochondrial dysfunction in neurons; (4) impaired synaptogenesis; (5) increased leakage and apoptosis in neurons; (6) brain neuropathology and impaired learning and memory in mice; and (7) Aβ peptide clearance and/or deposition, which determine their roles in the onset and progression of AD. Likewise, epidemiological studies have demonstrated that the ε4 allele was overrepresented in aMCI and that increased frequency of the allele was associated with an increased rate of cognitive decline, as well as the rate of conversion from MCI to AD^7–9^. Moreover, neuropathological studies suggest that the APOE ε4 status is associated with increased cerebrospinal fluid tau levels in cognitively healthy elders^10, 11^ and that these ε4-related differences were more pronounced in patients with MCI^11^. Collectively, these studies provide convergent evidence that the APOE ε4 allele typically increases the risk of progression from MCI to AD.

The combination of genetic assessment with neuroimaging is emerging as a promising preclinical AD research strategy. Compatible with the above-mentioned neuropathological findings, the synergistic effects of the APOE ε4 allele and MCI status on the brain’s structure and function were observed in several genetic imaging studies in which reductions of cerebral gray-matter volume^12^ and glucose metabolism^13^ were found in patients with MCI, and the extent of the reductions were exacerbated in MCI ε4 carriers^12, 14, 15^. It is worthy to note that AD has been considered as a disconnection syndrome^16^; thus, it is critical to understand alterations of the neuronal circuits underlying cognitive deficits in individuals at a high risk of AD. Numerous functional magnetic resonance imaging (fMRI)/magnetoencephalography (MEG)/electroencephagraphy (EEG) studies reported the alterations of the brain’s structural and functional connectivity in either patients with aMCI^17–20^ or healthy APOE ε4 carriers^21–25^, suggesting the association of the presence of aMCI or APOE ε4 status with the alterations of neuronal circuits. More importantly, a recent study^26^ used the MEG data to investigate the functional brain network at multiple frequency bands. In the low frequency band (delta and theta), interaction effects of diagnosis (i.e., MCI) and APOE genotype on functional connectivity strength were found in the frontal-temporal regions, indicating that the ε4 allele may induce changes in the network configuration with a different profile in healthy controls and MCI patients. Therefore, in the present study, we will use the resting-state (‘task-free’) functional magnetic resonance imaging data to construct the functional brain network and further investigate how the APOE genotype specifically modulates brain network connectivity in patients with aMCI.

Here, we employed resting-state functional magnetic resonance imaging (R-fMRI) to investigate the functional connectivity patterns of the whole-brain networks in 156 individuals including 66 aMCI patients (27 ε4 carriers and 39 non-carriers) and 90 healthy controls (45 ε4 carriers and 45 non-carriers). R-fMRI is a promising functional imaging technique that allows for the examination of the spontaneous or intrinsic functional connectivity patterns of the human brain (i.e., functional connectomics) in normal and diseased populations^27–30^. Recently, R-fMRI has been widely used to study functional brain networks in either healthy adults carrying APOE-ε4^22, 31^ or patients with aMCI^18, 19^. In the present study, we employed voxel-wise network centrality measure to capture the complexity of the functional connectome as a whole. This graph-based measure of network organization captures the function relationships of a given voxel (node) within the entire connectivity matrix of the brain (connectome), rather than with specific nodes or networks^32–34^. A variety of metrics index network centrality captures different aspects of connectivity, highlighting the importance of considering both local and global connectivity properties of the functional connectome^32^. We used two commonly employed measures, degree centrality (DC)^35, 36^ and eigenvector centrality (EC)^37–39^, to quantify local and global functional integrity of the brain connectome. Briefly, DC is a relatively local measure of the connectome graph that indexes the number of direct connections for a given node, whereas EC is a relatively global measure that indexes the qualitative superiority of a node’s connections, rather than the number of direct connections per se. Thus, examining voxel-wise DC and EC patterns allows us to investigate the diagnosis-by-genotype interactions on local and global information processing within the connectome without requiring selection of a prior nodes or network of interest. We hypothesize that the APOE ε4 is linked to a specific pattern of intrinsic functional disintegration of the brain networks in patients with aMCI.

## Materials and Methods

### Participants

Patients with aMCI and healthy controls were recruited to establish a registry at the Affiliated ZhongDa Hospital, Southeast University^12, 24^. Currently, in this registry, 222 participants (87 aMCI patients and 135 cognitively healthy elders) were included; all of them were Chinese Han and right-handed. All participants were recruited through a normal community health screening and newspaper advertisements, and they underwent a standardized clinical interview, neuropsychological battery assessment, genetic screening and multi-modal brain MRI examinations (for details, see the below). All aMCI patients met the diagnostic criteria proposed by Petersen *et al.* ^40^, including (i) subjective memory impairment corroborated by the subject and an informant; (ii) objective memory performance documented by an auditory verbal learning test with a 20 min delayed recall (AVLT-DR) score less than or equal to 1.5 times the SD of age- and education-adjusted norms (the cutoff was ≤4 correct responses on 12 items for ≥8 years of education); (iii) the mini-mental state examination (MMSE) score was greater than or equal to 24 or the mattis dementia rating scale-2 (MDRS-2) score was more than 120; (iv) preserved activities of daily living; and (v) insufficient level to meet the Alzheimer’s Criteria of National Institute of Neurological and Communicative Disorders and Stroke and the Alzheimer’s Disease and Related Disorders Association (NINCDS-ADRDA). The healthy controls were required to have an MMSE score ≥26, MDRS-2 score >120, and AVLT-DR score >4 for subjects with 8 or more years of education. Participants were excluded from the study if they had a history of neurological or psychiatric illness, major medical illness, severe visual or hearing loss or gross structural abnormalities revealed by MRI images. All subjects were required to sign informed consent documents prior to the experiments tests. All the clinical tests were approved by the Research Ethics Committee of Affiliated ZhongDa Hospital and the Southeast University and were carried out in accordance with the approved guidelines.

In the present study, we selected the data of 156 elderly participants, including 66 patients with aMCI and 90 healthy controls (HCs). Of these aMCI patients, there were 27 ε4 carriers (25 ε3/ε4 and 2 ε4/ε4) and 39 non-carriers (ε3/ε3); of these controls, there were 45 ε4 carriers (ε3/ε4) and 45 non-carriers (ε3/ε3). The participants with one or more ε2 allele(s) were excluded from this study due to the allele’s possible protective effects^41^. Notably, all participants included this study had no excessive motion artifacts (i.e., exceeding more than 3 mm translational movement or more than 3° rotational movement) during R-fMRI scan or incomplete image coverage. Table 1 presents the demographic information, APOE status and cognitive scores of the participants included in this study.

**Table 1:** Demographic and neuropsychological data for all participants.

Measure	HC		aMCI		*P*-values^a^		
	ε4 non-carriers (n = 45)	ε4 carriers (n = 45)	ε4 non-carriers (n = 39)	ε4 carriers (n = 27)	Diagnosis	Genotype	Interaction
**Demographics**							
Age (years)	68.6 ± 6.5	67.1 ± 6.4	68.1 ± 7.5	70.9 ± 7.2	0.146	0.550	0.051
Education (years)	12.3 ± 3.1	11.7 ± 2.8	12.1 ± 3.6	11.5 ± 3.1	0.757	0.233	0.963
Gender (male/female)	25/20	22/23	24/15	14/13	0.587	0.233	0.854
**Hippocampal Volume**							
Left Hippocampus	0.50 ± 0.04	0.52 ± 0.03	0.50 ± 0.06	0.47 ± 0.05	0.003	0.443	0.028
Right Hippocampus	0.47 ± 0.04	0.48 ± 0.03	0.47 ± 0.05	0.44 ± 0.05	0.011	0.110	0.018
**General cognition**							
MMSE	28.6 ± 1.0	28.3 ± 1.3	26.9 ± 1.9	26.0 ± 2.8	<0.001	0.064	0.564
MDRS-2 total (out of 144)	138.2 ± 2.7	138.1 ± 3.1	132.9 ± 5.2	130.1 ± 5.9	<0.001	0.068	0.135
Attention	36.27 ± 0.84	36.58 ± 0.58	36.10 ± 0.88	36.11 ± 0.93	0.036	0.128	0.378
Initiation/Preservation	36.80 ± 0.55	36.29 ± 1.67	34.54 ± 3.81	33.67 ± 3.63	<0.001	0.091	0.804
Construct	5.40 ± 0.81	5.47 ± 0.99	5.26 ± 0.82	5.44 ± 0.70	0.460	0.180	0.713
Conceptual	37.31 ± 1.31	37.38 ± 1.96	36.64 ± 1.72	36.52 ± 2.21	0.017	0.884	0.905
Memory	22.42 ± 1.45	22.40 ± 1.36	20.36 ± 2.07	18.37 ± 3.24	<0.001	0.004	0.013
**Composite** ***Z*** **scores of each cognitive domain**							
Episodic Memory	0.51 ± 0.56	0.55 ± 0.44	−0.58 ± 0.68	−0.93 ± 0.70	<0.001	0.217	0.165
AVLT-DR (raw score)	7.58 ± 1.95	7.67 ± 2.03	2.36 ± 1.61	1.78 ± 1.42	<0.001	0.570	0.630
LMT-DR (raw score)	8.54 ± 2.59	8.68 ± 2.50	5.26 ± 3.35	4.22 ± 3.40	<0.001	0.606	0.358
CFT-DR (raw score)	18.36 ± 6.06	18.63 ± 4.94	13.53 ± 6.09	9.63 ± 6.47	<0.001	0.103	0.110
Visuospatial Function	0.16 ± 0.54	0.32 ± 0.53	−0.06 ± 0.66	−0.72 ± 1.23	<0.001	0.115	0.004
CDT (raw score)	8.76 ± 1.21	8.91 ± 1.29	8.10 ± 1.71	7.48 ± 1.63	<0.001	0.558	0.251
CFT (raw score)	34.09 ± 1.76	34.62 ± 1.43	34.05 ± 1.75	31.81 ± 4.22	0.001	0.070	0.001
Information Processing Speed	0.32 ± 0.69	0.33 ± 0.81	−0.40 ± 0.68	−0.51 ± 0.73	<0.001	0.769	0.697
DSST (raw score)	38.98 ± 10.48	39.84 ± 10.51	30.41 ± 9.63	28.52 ± 9.56	<0.001	0.698	0.995
TMT-A (raw score, second)	66.04 ± 17.89	64.16 ± 17.15	81.85 ± 26.65	86.78 ± 47.91	<0.001	0.880	0.819
Stroop A (raw score, second)	26.24 ± 4.84	26.93 ± 6.79	31.46 ± 7.50	33.78 ± 8.90	<0.001	0.378	0.966
Stroop B (raw score, second)	39.07 ± 9.11	41.44 ± 12.32	47.64 ± 11.02	47.67 ± 13.55	<0.001	0.621	0.242
Executive Function	0.30 ± 0.56	0.30 ± 0.66	−0.35 ± 0.55	−0.50 ± 0.60	<0.001	0.880	0.945
VFT-objects (raw score)	26.71 ± 4.91	25.18 ± 5.96	20.59 ± 6.26	19.33 ± 6.31	<0.001	0.134	0.602
VFT-animals (raw score)	21.98 ± 5.81	20.64 ± 4.85	16.95 ± 4.03	15.78 ± 4.91	<0.001	0.320	0.519
DST-backward (raw score)	4.78 ± 1.36	5.29 ± 1.52	4.36 ± 1.31	4.44 ± 1.45	0.016	0.065	0.588
TMT-B (raw score, second)	179.44 ± 62.83	178.11 ± 52.56	243.08 ± 100.87	279.93 ± 135.90	<0.001	0.409	0.630
Stroop C (raw score, second)	78.36 ± 20.17	81.93 ± 26.27	102.13 ± 28.73	94.67 ± 24.80	<0.001	0.388	0.031
Similarity (raw score)	19.00 ± 3.40	19.69 ± 2.41	17.95 ± 3.15	16.19 ± 4.39	<0.001	0.591	0.052

Data are presented as the mean ± standard deviation (SD).

^a^ *P*-values were obtained by two-way analysis of covariance (ANCOVA). The performance of each neuropsychological test is expressed as raw scores. The level of each cognitive domain is denoted by the composite *Z* scores.

“ε4 carriers” denotes the subjects who possessed at least one APOE ε4 allele; “ε4 non-carriers” denotes the subjects who possessed homozygous for the APOE ε3 allele.

Abbreviations: aMCI, amnestic mild cognitive impairment; HC, healthy control; MMSE, mini-mental state examination; MDRS-2, Mattis dementia rating scale-2; AVLT-DR, auditory verbal learning test-20 min delayed recall; LMT-DR, logical memory test-20 min delayed recall; CFT-DR, Rey-Osterrieth complex figure test-20 min delayed recall; CDT, clock drawing test; CFT, Rey-Osterrieth complex figure test; DSST, digital symbol substitution test; TMT-A, trail making test-A; Stroop, Stroop color test; VFT, verbal fluency test; DST, digit span test; TMT-B, trail making test-B; Similarity, semantic similarity test.

### Neuropsychological Assessment

For all participants, we assessed their general cognitive function using MMSE and MDRS-2, and performed a neuropsychological battery to evaluate their specific functions in episodic memory, visuospatial skills, information processing speed and executive function, respectively. This battery consisted of the AVLT-DR, the logical memory test with a 20 min delayed recall, the Rey-Osterrieth complex figure test with a 20 min delayed recall, the clock drawing test, the digital symbol substitution test, trail-making test A and B, the stroop color-word test A, B and C, the verbal fluency test, the digital span test and the semantic similarity test.

In this study, we performed a composite score analysis^24^ of these neurocognitive measures to increase statistical power by reducing random variability and floor and ceiling effects. Briefly, for each subject, the raw scores from each test were first transformed to *z* scores with reference to the means and standard deviations of the test across all subjects. Then, the composite scores were calculated by averaging the *z* scores within the neuropsychological domains listed below: episodic memory (3 tests, including the AVLT-DR, the logical memory test with a 20 min delayed recall, and the Rey-Osterrieth complex figure test with a 20 min delayed recall), visuospatial function (2 tests, including the Rey-Osterrieth complex figure test and the clock drawing test), information processing speed (4 tests, including the digital symbol substitution test, the trail making test-A, and Stroop A and B) and executive function (5 tests, including the verbal fluency test, the digital span test-backward, the trail making test-B, Stroop C and the semantic similarity test). Notably, MMSE and MDRS-2 were used for descriptive and diagnostic classifications, but not for the composite measures.

### APOE Genotyping

Genomic DNA of each subject was extracted from 250 µl EDTA-anticoagulated blood using a DNA direct kit (Tiangen, China). A polymerase chain reaction-based restriction fragment length polymorphism (PCR-RFLP) assay was applied to detect the alleles of rs7412 and rs429358, the haplotype of which ultimately determined the APOE genotype. The specific process was described in the Supplementary Information.

### Data Acquisition

MRI images were acquired in a 3.0 T Siemens Verio scanner (Siemens, Erlangen, Germany) with a 12-channel head coil at the Affiliated ZhongDa Hospital of Southeast University. All participants lay supine with the head snugly fixed by a belt and foam pads to minimize head movement. High-resolution T1-weighted axial images covering the whole brain were acquired using 3D magnetization prepared rapid gradient echo (MPRAGE) sequence as below: repetition time (TR) = 1900 ms; echo time (TE) = 2.48 ms; flip angle (FA) = 9°; acquired matrix = 256 × 256; field of view (FOV) = 250 mm × 250 mm; thickness = 1.0 mm; gap = 0 mm; and number of slices = 176. Resting-state functional images were obtained for eight minutes with gradient-recalled echo-planar imaging (GRE-EPI) sequence: TR = 2000 ms; TE = 25 ms; FA = 90°; acquisition matrix = 64 × 64; FOV = 240 mm × 240 mm; thickness = 4.0 mm; gap = 0 mm; and number of slices = 36. Prior to the scan, all subjects were instructed to keep their eyes closed but not fall asleep, relax their minds, and move as little as possible during data acquisition. A simple questionnaire indicated that all of the subjects had not fallen asleep during the scan.

### Data Preprocessing

Data preprocessing was carried out using Statistical Parametric Mapping (SPM8, http://www.fil.ion.ucl.ac.uk/spm) and the Data Processing Assistant for Resting-State fMRI (DPARSF, http://www.restfmri.net/forum/dparsf). The first ten functional volumes were discarded for scanner stabilization and participants’ adaption to the circumstances. The remaining images were corrected for timing differences and motion effects. Next, the individual structural images (T1-weighted MPRAGE images) were co-registered to the mean functional image after motion correction using a linear transformation. The transformed structural images were then segmented into gray matter, white matter and cerebrospinal fluid using a unified segmentation algorithm^42^. The motion corrected functional volumes were spatially normalized to the Montreal Neurological Institute space and resampled to 3 mm isotropic voxels using the normalization parameters estimated during unified segmentation. Further preprocessing included linear de-trending and temporal band-pass filtering (0.01–0.1 Hz), which were applied to reduce the effects of low-frequency drift and high-frequency physiological noise^27, 43^. Finally, the nuisance signals (six head motion parameters, mean global signal, white matter signal and cerebrospinal fluid signal) were extracted and regressed out from the data. Given that the removal of the global signal introduced a shift in the distribution of correlation coefficients (mainly the presence of negative correlations) and made biological interpretation ambiguous^44, 45^, we restricted our explorations to positive correlations, as in previous studies^35, 46^.

### Network Analysis

We performed whole-brain network centrality analysis using the GRETNA package (http://www.nitrc.org/projects/gretna/)^47^. Briefly, for each subject, we computed the Pearson’s correlations between the time series of all pairs of brain voxels, resulting in a whole-brain functional connectivity matrix. This computation was constrained within a gray matter mask (*N*_*voxels*_ = 57,641), which was generated by setting a threshold of 0.2 on the mean gray matter probability map across all subjects. Then, we performed weighted degree centrality (DC) and eigenvector centrality (EC) analyses in a voxel-wise manner to quantify local and global functional integrity of the brain networks. (i) *Degree centrality analysis*. For a given gray matter voxel, *i*, we computed its DC using the following equation:

1 $$DC(i)=\frac{1}{N-1}\sum _{j\ne i}{z}_{ij}{r}_{ij} > {r}_{{0}}$$

where *r*_*ij*_ was the correlation coefficient between voxel *i* and voxel *j*, *r*_*0*_ was a threshold that was set to eliminate weak correlations possibly arising from signal noise (*r*_*0*_ = 0.2 in this study), and *r*_*ij*_ was converted to *z*_*ij*_ using Fisher’s *Z*-transformation when calculating DC. The brain voxels with higher DC values usually indicate their central roles in the local functional integrity of the whole-brain networks. (ii) *Eigenvector centrality analysis*. The EC is simply the first eigenvector of the connection matrix, which corresponds to the largest eigenvalue *λ*_1_:

2 $$EC(i)={\mu }_{1}(i)=\frac{1}{{\lambda }_{1}}A{\mu }_{1}=\frac{1}{{\lambda }_{1}}\sum _{j=1}^{N}{r}_{ij}{\mu }_{1}(j)$$

where *r*_*ij*_ was the correlation coefficient between voxel *i* and voxel *j*, A was the connection matrix, *λ*_1_ was the largest eigenvalue of connection matrix and *μ*_1_ was the corresponding eigenvector. The brain voxels with higher EC values are usually strongly correlated with many other nodes that are themselves central within the network and indicate their central roles in the global functional integrity of the whole-brain networks. As a result, for each subject, we obtained individual DC and EC maps for further statistical analysis.

### Statistical Analysis

#### Demographic and Neuropsychological Variables

Statistical analyses of demographics and cognitive performance were performed using two-way analysis of covariance (ANCOVA) for continuous variables and using chi-square tests for categorical variables. Specifically, the main effects of diagnosis (aMCI vs. HC) and APOE genotype (ε4 carriers vs. non-carriers), and diagnosis-by-genotype interactions were assessed. For the ANCOVA analyses in the cognitive variables, age, gender and years of education were considered as unconcerned, confounding factors. These analyses were implemented in SPSS 17.0 (SPSS, Inc., Chicago, IL).

#### Group Differences in Brain Network Connectivity

Before statistical analysis, all individual DC and EC maps were spatially smoothed with a Gaussian kernel (full width at half-maximum [FWHM] = 6 mm). A voxel-wise two-way ANCOVA was separately performed to examine the main effects of diagnosis (aMCI vs. HC) and APOE genotype (ε4 carriers vs. non-carriers), and the diagnosis-by-genotype interactions on DC and EC maps, with age, gender and year of education as covariates. This analysis was implemented using the SurfStat (http://www.math.mcgill.ca/keith/surfstat/) and correction for multiple comparisons was conducted by Monte Carlo simulations using the AFNI AlphaSim program (http://afni.nimh.nih.gov/pub/dist/doc/manual/AlphaSim.pdf). The *α* level of 0.05 was obtained with a voxel-wise *P* < 0.05 and cluster size >4,266 mm^3^. Once there were regions showing significant diagnosis-by-genotype interactions, post-hoc general linear model analyses were further performed to examine connectivity differences between the ε4 carriers and non-carriers in the aMCI group and in the HC group, separately, with age, gender and year of education as unconcerned, confounding factors.

#### Relationship Between Network Connectivity and Cognitive Variables

We performed multiple linear regression analyses to examine the relationships between the neuropsychological composite *Z* scores (i.e., episodic memory, visuospatial function, information processing speed and executive function) and network centrality values (i.e., DC and EC) in brain areas showing significant diagnosis-by-genotype interactions. These analyses were separately conducted in each subgroup, with age, gender and years of education as unconcerned, confounding factors.

### Validation Analysis

We evaluated whether our main results were influenced by several confounding factors (e.g., the gray matter atrophy, connectivity threshold, head motion and potentially artificial local correlations). The detailed validation analyses are described in Supplementary Information.

## Results

### Demographic and Neuropsychological Variables

Table 1 illustrates the demographics and neuropsychological measures for aMCI and HC participants stratified by APOE ε4 status. The four subgroups did not differ in age, gender and years of education (all *Ps* > 0.05). Two-way ANCOVA analyses revealed the main effects of diagnosis and APOE genotype and the diagnosis-by-genotype interactions on neurocognitive measures. Briefly, a significant main effect of diagnosis on each cognitive domain was observed, with the aMCI group showing worse cognitive performance than the HC group. There was no significant main effect of APOE genotype on any cognitive measure. Notably, we observed a significant interaction between diagnosis and APOE genotype only on visuospatial function, with ε4 carriers showing worse performance than non-carriers in the aMCI group but no genotype difference in the HC group.

### Group-based Differences in Brain Network Centrality

#### Degree Centrality

**Figure 1:**
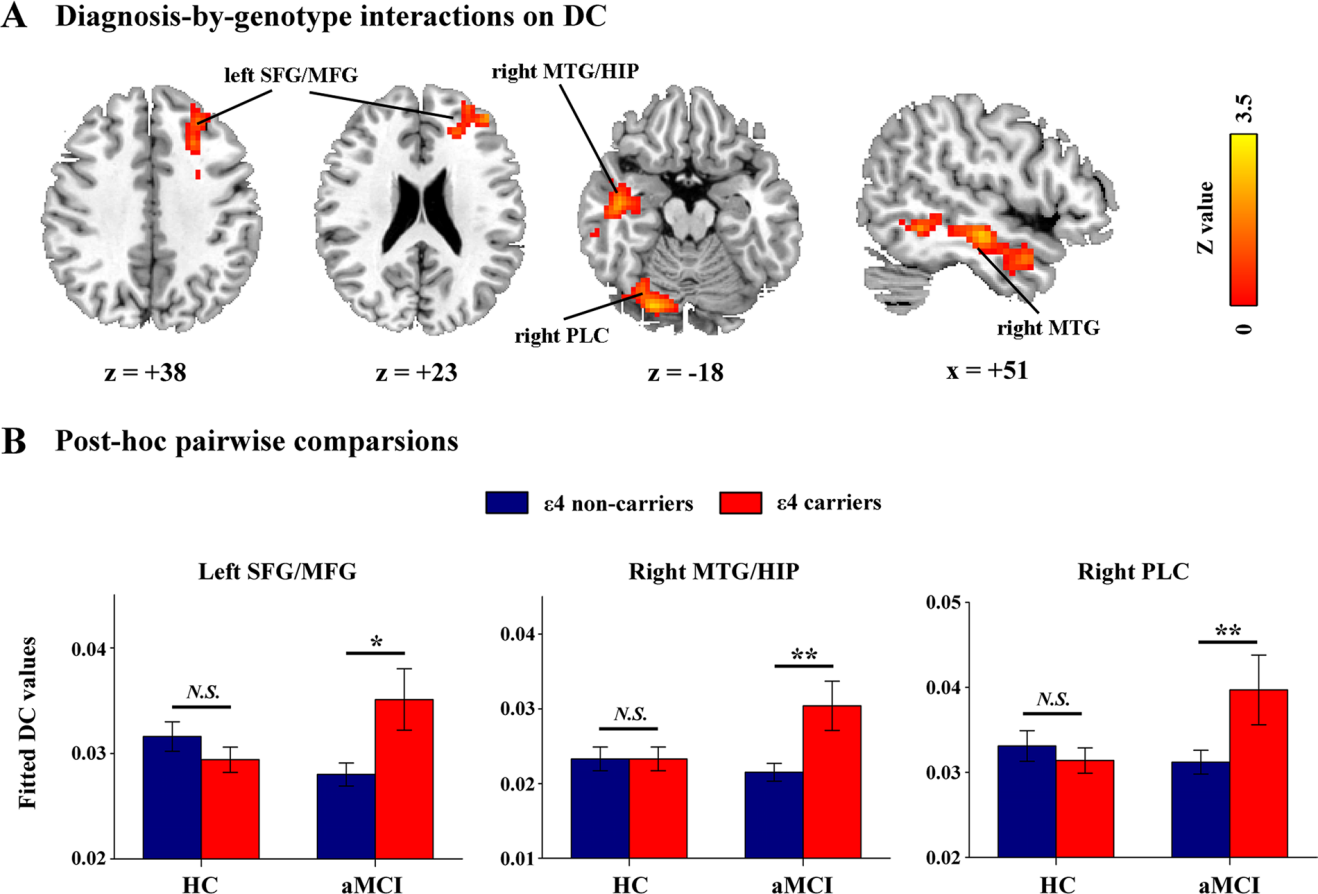
Diagnosis-by-genotype interactions on DC. (**A**) Two-way ANCOVA revealed significant diagnosis-by-genotype interactions on DC in the left superior/middle frontal gyrus (SFG/MFG), right middle temporal gyrus (MTG) extending to the hippocampus (HIP), and right posterior lobe of the cerebellum (PLC). The color bar indicates the statistical significance threshold (*Z*-score). Multiple comparisons were performed by a combined |*z*| > 1.96 (*P* < 0.05) and cluster size > 4,266 mm^3^, which corresponded to a corrected *P* < 0.05. (**B**) The bar graphs illustrate post-hoc pairwise comparisons in the regions showing significant diagnosis-by-genotype interactions. The differences between the ε4 carriers and non-carriers were significant in the aMCI group but not in the HC group. The data were expressed as the mean (M) ± standard error (SE). DC, degree centrality; HC, healthy control; and aMCI, amnestic mild cognitive impairment. *N.S.*, Non-significant. *P < 0.05, **P < 0.01.

The spatial patterns of whole-brain DC maps in the four subgroups were very similar by visual inspection (Figure S1). Statistical analyses revealed non-significant main effects of either diagnosis or APOE genotype and significant diagnosis-by-genotype interactions on DC in the left superior and middle frontal gyrus (SFG/MFG, including BA9, BA10 and BA46 areas), right middle temporal gyrus (MTG) extending to the hippocampus (HIP) and right posterior lobe of the cerebellum (PLC, the junction of right lobule VI/Crus I) (Table 2, Fig. 1A). Post-hoc pairwise analysis further revealed that these regions exhibited significantly higher DC values in the ε4 carriers than the non-carriers in the aMCI group, but no genotype difference was observed in the HC group (Fig. 1B).

**Table 2:** Diagnosis-by-genotype interactions on degree centrality and eigenvector centrality.

Cluster Regions	BA	Cluster Size (voxels)	*Z* value	MNI coordinates (Peak)		
				*x*	*y*	*z*
**Degree Centrality**						
Right MTG/HIP	21	417	3.150	51	−18	−12
Left SFG/MFG	9/10/46	524	3.078	−36	3	60
Right PLC (lobule VI/Crus I)	—	379	3.213	24	−81	−18
**Eigenvector Centrality**						
Right MTG	21	184	3.770	51	−18	−12
Bilateral vACC/vMPFC	11	576	3.532	6	36	−18
Bilateral RSC	29	206	2.939	−12	43	3

Abbreviations: MTG, middle temporal gyrus; HIP, hippocampus; SFG, superior frontal gyrus; MFG, middle frontal gyrus; PLC, posterior lobe of cerebellum; vACC/vMPFC, ventral anterior cingulate/ventral medial prefrontal cortex; RSC, retrosplenial cortex; BA, Brodmann area; and MNI, Montreal Neurological Institute.

#### Eigenvector Centrality

**Figure 2:**
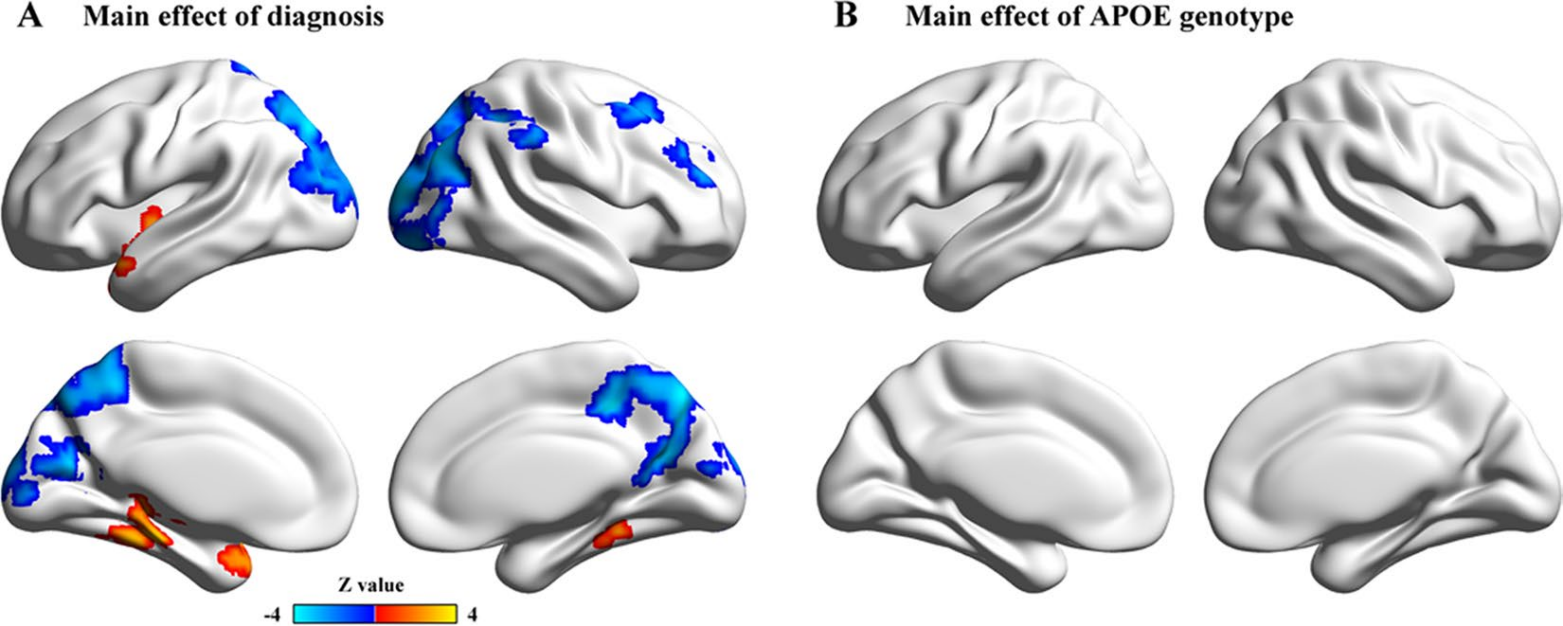
Main effect of diagnosis on EC. (**A**) Statistical map of the main effects of diagnosis. The statistical significance threshold was set at |*z*| > 1.96 (*P* < 0.05), and cluster size > 4,266 mm^3^, which corresponded to a corrected *P* < 0.05. The color map shows significant differences in *Z* between the aMCI and HC groups. Warm colors represent higher EC values in the aMCI group compared with the HC group, while the cool colors represent the opposite. (**B**) Statistical map of the main effects of APOE genotype. There was no significant effect of APOE genotype on EC. EC, eigenvector centrality; HC, healthy control; and aMCI, amnestic mild cognitive impairment.

**Figure 3:**
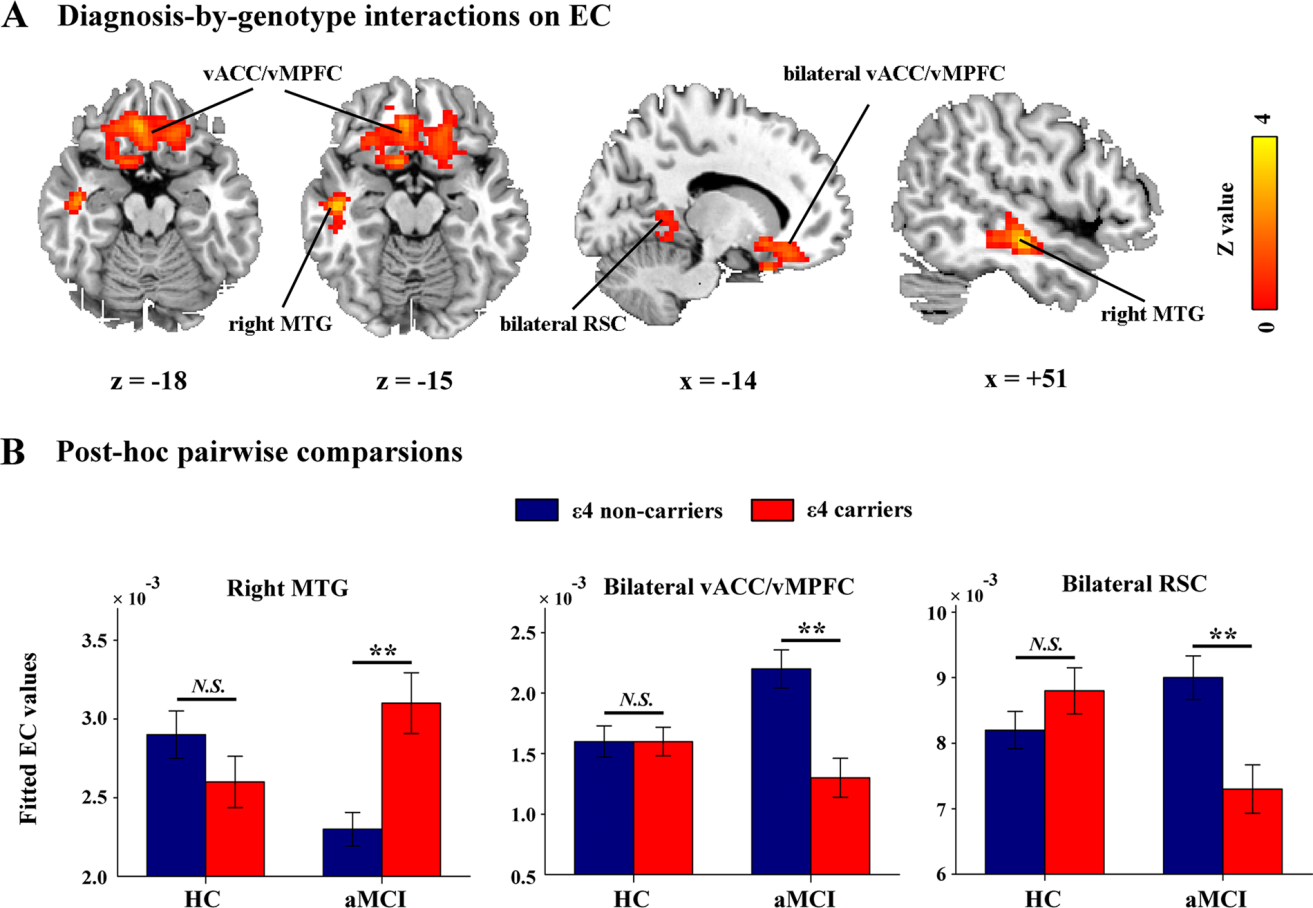
Diagnosis-by-genotype interactions on EC. (**A**) Two-way ANCOVA revealed significant diagnosis-by-genotype interactions on EC in the right middle temporal gyrus (MTG), bilateral ventral anterior cingulate/ventral medial prefrontal cortex (vACC/vMPFC) and retrosplenial cortex (RSC). The color bar represents the statistical significance threshold (*Z*-score). Multiple comparisons were conducted by a combined |*z*| > 1.96 (*P* < 0.05) and cluster size > 4,266 mm^3^, which corresponded to a corrected *P* < 0.05. (**B**) The bar graphs depict post-hoc pairwise comparisons in the regions showing significant diagnosis-by-genotype interactions. The differences between the ε4 carriers and non-carriers were significant in the aMCI group but not in the HC group. The data were expressed as the mean (M) ± standard error (SE). EC, eigenvector centrality; HC, healthy control; and aMCI, amnestic mild cognitive impairment. *N.S.*, Non-significant. **P < 0.01.

The spatial patterns of whole-brain EC maps were also similar among the four subgroups (Figure S2). The main effects of diagnosis and APOE genotype on EC were illustrated in Fig. 2. Compared with the HCs, the patients with aMCI exhibited lower EC values primarily in the posterior cingulate cortex/precuneus, right middle frontal gyrus, bilateral intraparietal cortex and bilateral visual cortex and higher EC values in the left insula, left superior temporal gyrus and the left parahippocampal gyrus (Fig. 2A). No regions showed significant main effects of APOE genotype (Fig. 2B). Importantly, significant diagnosis-by-genotype interactions on EC were found in the right MTG, bilateral ventral anterior cingulate/ventral medial prefrontal cortex (vACC/vMPFC) and bilateral retrosplenial cortex (RSC) (Table 2, Fig. 3A). Post-hoc pairwise analysis revealed that compared with the non-carriers, the ε4 carriers had significantly higher EC values in the right MTG and lower EC values in the vACC/vMPFC and RSC in the aMCI group, but there were no significant genotype differences in the HC group (Fig. 3B).

### Relationship Between Network Centrality Measures and Neurocognitive Variables

**Figure 4:**
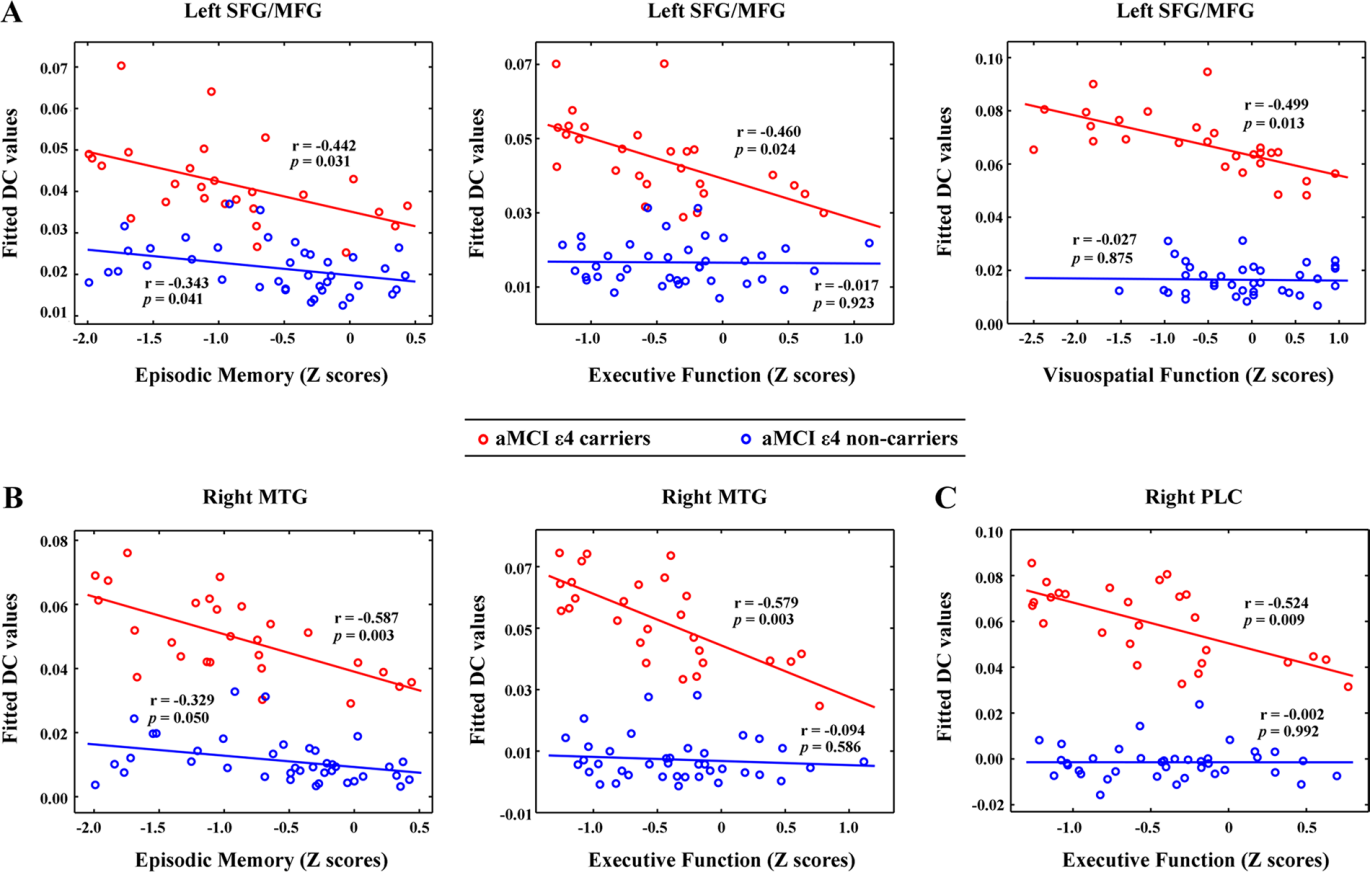
Relationship between the cognitive performance and regional DC values in the aMCI patients. The scatter plots showed correlations between the cognitive performance and regional DC values in the left SFG/MFG (**A**), right MTG (**B**) and right PLC (**C**) in the aMCI ε4 carriers (red circles) and the aMCI ε4 non-carriers (blue circles). Note that no significant correlations were observed between the DC values in these regions and cognitive performances in the HC ε4 carriers and HC ε4 non-carriers (all *Ps* > 0.05). DC, degree centrality; aMCI, amnestic mild cognitive impairment; HC, healthy control; SFG/MFG, superior/middle frontal gyrus; MTG, middle temporal gyrus; and PLC, posterior lobe of the cerebellum.

We found that the DC values in the left SFG/MFG, right MTG/HIP and right PLC were negatively correlated with the cognitive performance (e.g., episodic memory, executive function and visuospatial function) in the aMCI ε4 carriers (Fig. 4A–C, red circles). The DC values in the left SFG/MFG were also negatively correlated with episodic memory in the aMCI ε4 non-carriers (Fig. 4A, left panel, blue circles). However, no significant correlations between the DC values in these regions and cognitive performance were observed in either the ε4 carriers or non-carriers in the HC group. Furthermore, there were no significant correlations between EC values and cognitive measures in each subgroup.

### Validation Results

In general, we found that our main results were reproducible after considering the effects of gray matter atrophy (Supplementary Information Figure S4), connectivity thresholds (Supplementary Information Figure S5), head motion (Supplementary Information Figure S6) and potentially artificial local correlations (Supplementary Information Figure S7). The detailed validation results are described in Supplementary Information.

## Discussion

Using R-fMRI and network centrality approaches, we showed that the APOE genotype has disease-specific effects on brain functional integration both at the local and global level. Specifically, both DC and EC showed ε4-related increases in centrality within the right lateral temporal cortex (i.e., MTG) in the aMCI group. Moreover, in the aMCI group, DC showed ε4-related increases in network centrality within the left prefrontal cortex and right cerebellar cortex, while EC showed ε4-related decreases in centrality within the bilateral vACC/vMPFC and RSC. However, these genotype differences were absent in the healthy controls. These findings suggest that the APOE-ε4 allele is linked to a specific pattern of intrinsic functional disintegration of the brain in aMCI patients, facilitating our understanding of how the entire assembly of the brain network reorganizes in response to APOE variants in aMCI.

### Diagnosis-by-Genotype Interactions on Network Centrality

Combining genetic risk with sensitive functional brain imaging technologies may augment our ability to detect individuals that are likely to develop AD before actual disease onset. In the present study, the ANCOVA analyses (diagnosis-by-genotype) revealed that the deficits of brain function related to APOE-ε4 status were detected in the condition involving a synergistic interaction with aMCI status, suggesting that pathological status may enhance the effect of the risk genotype on the brain functional architecture. It is possible that in patients with aMCI, the risk locus directly influences gene expression or splicing, promoting linkage disequilibrium with another variant that has the same influence, such as microtubule-associated protein tau-H1 genetic variants^48^. It is also possible that this risk is conferred by some other mechanisms^49^.

In the present study, we first observed ε4-related increases in network centrality in the right MTG in aMCI patients, both for DC and EC. This indicates that the right MTG may act as a pivotal incoming or outgoing hub within the brain network, maintaining information flow, both at local and global level in aMCI ε4 carriers. Episodic memory involves lateral temporal cortex as part of its neural substrate^50^, and a micro-electrode study reported that neurons with changes related to recognition retrieval were more frequent in the superior part of MTG and the superior temporal gyrus^51^. Neuroimaging studies also demonstrated that the MTG is associated with cued attention and working memory^52, 53^. Previous fMRI studies showed increased activation or functional connectivity in the MTG of AD patients^53^ or individuals at high-risk of AD^54, 55^. Therefore, compatible with these studies, the increased intrinsic network centrality within the MTG in the aMCI ε4 carriers may be interpreted as reflecting greater cognitive “effort” by aMCI ε4 carriers to achieve the same level of performance (e.g., episodic memory) as aMCI ε4 non-carriers. Moreover, it is demonstrated that highly connected hub regions are particularly vulnerable to AD pathology (e.g., Aβ deposition)^35^. Decreased network centrality in the MTG or a loss of hub regions in the temporal lobe were observed in AD patients^56, 57^. Therefore, increased network centrality within the MTG in aMCI ε4 carriers may be indicative of premorbid functional weakness and herald the presence of further AD. Longitudinal studies are needed to assess whether increased network centrality within the MTG foreshadow clinical decline.

Second, in the aMCI group, but not in the HC group, we also observed ε4-related DC increases in centrality within the left prefrontal cortex including the BA9, BA10 and BA46 areas and right PLC (i.e., the junction of right lobule VI/Crus I), suggesting a more prominent role of the prefrontal cortex and cerebellum in coordinating the functional brain network at the local level in aMCI ε4 carriers. It is demonstrated that the rostral prefrontal cortex (BA10) is involved in memory storage and retrieval^58^, and the dorsolateral prefrontal cortex (BA 9 and BA 46) is mainly involved in the executive control process^59, 60^. Moreover, previous neuroimaging studies revealed that the cerebellum has extensive reciprocal connections with cerebral cortex and the limbic system, and demonstrated that the lobules VI and VII (including Crus I and II) contribute to higher-level processes, such as language, working memory, executive function, and attention^61^. It is noteworthy that episodic memory and executive function, which are specifically impaired in AD/MCI, were relatively preserved in the aMCI ε4 carriers compared with the aMCI ε4-noncarriers. Therefore, one possible explanation is that this hyper-functional connectivity in the aMCI ε4 carriers represents the brain’s attempt to maintain the same cognitive performance as aMCI ε4 non-carriers. Support for this compensation mechanism has been provided by previous neuroimaging studies in AD/AD-risk^53, 62–64^. Moreover, the striking correlations between regional DC values in the prefrontal cortex and PLC with the cognitive performance (i.e., episodic memory and executive function *Z* scores) in the aMCI ε4 carriers further suggested that increased functional connectivity in these regions may attempt to bolster the stabilization of the functional network. However, we also presumed that the underlying deficits would begin to surface only when this compensatory mechanism becomes overextended.

Finally, we observed ε4-related EC decreases in several key nodes within the default-mode network (DMN) regions (i.e., bilateral RSC and vACC/vMPFC) in the aMCI group but not in the HC group. This finding suggests a diminished role of these regions in global network function in aMCI ε4 carriers. As a crucial transition region between the posterior cingulate cortex and posterior hippocampus, the RSC is known to have strong reciprocal afferent and efferent connections with the medial temporal lobe (e.g., entorhinal cortex and hippocampus) and has therefore been implicated in memory function^65, 66^, which is specifically impaired in AD/aMCI. More recently, neuroimaging studies suggested that spatial orientation is likely to be contingent upon the preservation of the RSC^67^. Thus, we speculated that visuospatial function deficits observed in aMCI ε carriers may be partly attributed to the dysfunction of RSC (i.e., decreased EC in the RSC), though no significant correlations between regional EC values in the RSC and the cognitive performance were observed. Indeed, neurodegenerative changes in the RSC, such as hypometabolism and atrophy, have been identified in early AD^13, 68^, even in individuals at high risk of AD^69^. Moreover, a recent study^70^ found that the RSC failed to deactivate in aMCI and AD during a word list-learning task and that more extensive impairment of the RSC was significantly related to smaller entorhinal and hippocampal volume, indicating that RSC dysfunction is an early characteristic of prodromal AD. The vACC/vMPFC is another area of predilections for AD. Evidence from fMRI studies has demonstrated that vACC/vMPFC has wide connections with the medial temporal lobe (e.g., hippocampus)^71, 72^. Numerous studies in early AD have reported increased amyloid load and hypometabolism in this region^73–76^. Further, hypometabolism in the vACC/vMPFC was found to distinguish aMCI converters from non-converters^77^. Compatible with our findings, a recent study observed greater amyloid deposition in the bilateral vACC/vMPFC in early MCI ε4 carriers than non-carriers^78^. Overall, in the aMCI patients, we demonstrated ε4-related decreases in EC within the bilateral RSC and vACC/vMPFC, indicating their diminished roles of coordinating the functional brain network at the global level, presumably in response to AD pathology.

More importantly, we observed opposite effects of the APOE-ε4 genotype on network centrality in aMCI patients (i.e., increased DC and decreased EC in aMCI ε4 carriers). Twamley *et al.* ^79^ reviewed studies of preclinical AD and proposed a nonlinear trajectory of episodic memory decline in which there is a long period of lowered but stable memory capacity in individuals with preclinical AD – perhaps due to neural compensatory mechanisms – that is followed by a relatively precipitous decline in the period immediately preceding the development of overt dementia. Importantly, Bajo *et al.* ^80^ recently found with MEG increased functional connectivity among temporoparietal regions in progress MCI patients as a sign of compensatory mechanism for the inefficiency of the memory networks. In this case, our present study is compatible with previous studies and suggests that increased local connectivity could be a result of counterbalancing APOE ε4-related disruption of global functional integrity in patients with aMCI. Further longitudinal studies are warranted to examine whether DC- and EC-related changes in these AD-related regions would imply AD conversion for aMCI ε4 carriers.

### Main Effects of the aMCI and APOE Genotype Status on Network Centrality

Regardless of the APOE genotype, EC revealed aMCI-related decreases in network centrality within the posterior cingulate cortex/precuneus, right middle frontal gyrus, and bilateral intraparietal cortex, most of which are components of the DMN^35, 81, 82^. Aberrant DMN activity and functional connectivity have been observed in AD/aMCI^46, 83, 84^; importantly, the disrupted DMN activity and connectivity could distinguish AD from healthy aging with high sensitivity and specificity^46, 83^. Therefore, previous studies and our present study support the idea that DMN regions comprise the typical predilection sites of AD and indicate that DMN connectivity may prove to be a sensitive and specific biomarker for AD. EC also revealed aMCI-related decreases in centrality within the occipital cortex, including the calcarine fissure, cuneus, and lingual gyrus. Previous studies in AD have reported reduced activity in the lingual gyrus^85^ and hypometabolism in the calcarine fissure^86^. Additional evidence from aMCI studies has shown demyelination in the lingual gyrus^87^. Our findings are highly compatible with these results. It is noteworthy that DC did not reveal any aMCI-related differences in network centrality, suggesting the preservation of local organization in aMCI patients. Together with DC and EC analyses, these findings suggest that global network integrity may be more preferentially affected in patients with aMCI. Finally, the present ANCOVA analyses revealed no significant main effect of the APOE genotype on brain network connectivity (either DC or EC) in the resting-state brain, although previous genetic imaging studies in healthy subjects have suggested an association between the APOE ε4 allele and the functional architecture of the brain^21–23^. The apparent lack of a clear effect in this present study may be due to the heterogeneity of participants: combining the healthy subjects and aMCI patients together in the ANCOVA analysis may reduce the ability to detect the effect of the APOE genotype on brain functional connectivity. A recent arterial spin labeling study^88^ reported an opposite effect of APOE genotype on regional cerebral perfusion in healthy subjects and patients with MCI, which may partly support our speculation.

### Further Considerations

Several issues need to be addressed. First, aMCI patients exhibit different progressive trajectories, where some ultimately develop AD and others do not. Accordingly, further follow-up longitudinal studies are warranted to examine whether the combination of brain network connectivity measures with the APOE genotype would improve the prediction of the conversion from aMCI to AD. Second, the present study did not examine the ε2-related effects on brain network connectivity due to a small sample size; thus, further studies including ε2 carriers would be important to expand upon these preliminary findings. Third, many previous studies revealed that brain structure and function could be influenced by additional gene variants^48, 89, 90^; thus, further studies that focus on more complex haplotypes are necessary and important to examine gene-gene interactions on brain network topology. Finally, a considerable amount of clinical and biological heterogeneity existed in the present sample of MCI participants whose recruitment was based on the clinical criteria only. This limitation could be overcome by adding neuropathological biomarkers to better characterize the study groups. However, a neurodegeneration biomarker as the hippocampal atrophy measured by T1 MRI images was obtained in the present study. So, according to the new guidelines for the MCI AD-related diagnosis^91, 92^, the MCI patients showing hippocampal volume reduction in comparison to the healthy controls would fulfill the MCI due to AD-intermediate likelihood diagnosis.

## Electronic supplementary material

Supplementary Information
