# Supplementary material for: APOE Genotype Effects on Intrinsic Brain Network Connectivity in Patients with Amnestic Mild Cognitive Impairment
Source: Sci Rep. 2017 Mar 24;7:397. doi: 10.1038/s41598-017-00432-0 (PMC5428452; doi:10.1038/s41598-017-00432-0)
Supplement: Supplementary file 1 — Supplementary Information [file 41598_2017_432_MOESM1_ESM.doc]

**APOE Genotype Effects on Intrinsic Brain Network Connectivity in Patients with Amnestic Mild Cognitive Impairment**

Zan Wang^1#^, Zheng-jia Dai^2, 3#^, Hao Shu^1^, Xu-hong Liao^2^, Chun-xian Yue^1^, Duan Liu^1^, Qi-hao Guo^4^, Yong He^2^* and Zhi-jun Zhang^1^*

^1^ Department of Neurology, Affiliated ZhongDa Hospital, School of Medicine, Southeast University, Nanjing, Jiangsu 210009, China

^2^ Department of Psychology, Sun Yat-sen University, Guangzhou 510006, China

^3^ State Key Laboratory of Cognitive Neuroscience and Learning & IDG/McGovern Institute for Brain Research, Beijing Normal University, Beijing 100875, China

^4^ Department of Neurology, Huashan Hospital, Fudan University, Shanghai 200040, China.

^#^ Zan Wang and Zheng-jia Dai contributed equally to this work.

**Supplemental Materials and Methods**

**APOE Genotyping**

A polymerase chain reaction-based restriction fragment length polymorphism (PCR-RFLP) assay detected the alleles of rs7412 and rs429358, respectively. The amplification reaction system contained 1 × GC buffer I (TAKARA), 2.0-mM Mg2+, 0.2-mM dNTP, 1 unit HotStarTaq polymerase (Qiagen Inc.), 1 µl genomic DNA and 1 µl (2 µM) primer for each allele (for rs429358, forward primer: AGGGCGCTGATGGACGAGAC, reverse primer: GCCCCGGCCTGGTACACT; for rs7412, forward primer: GGCGCGGACATGGAGGAC, reversed primer: GCCCCGGCCTGGTACACT). PCR cycling conditions were set as follows: (1) 95ºC for 15 min; (2) we performed 11 cycles, each of which included (i) 94ºC for 20 s, (ii) maintenance of the temperature at 0.5 ºC below the melting temperature for 40 s, (iii) 72ºC for 1 min and 40 s; (3) we performed another 24 cycles, each of which included (i) 94ºC for 20 s, (ii) maintenance of the temperature at 6ºC below the melting temperature for 40 s, (iii) 72ºC for 1.5 min; (4) 72ºC for 2 min. Amplification was carried out on a 2720 Thermal Cycler (ABI). Then, 10 µl amplified product was digested with 1 unit restriction endonuclease (AflⅢ for rs429358 and HaeⅡ for rs7412) at 37ºC overnight. Finally, the digested product was diluted tenfold and analyzed by capillary electrophoresis to detect the alleles of rs429358 and rs7412. As a result, the APOE genotype was determined by the haplotype of rs429358 and rs7412. The APOE ε2 allele was recognized by rs429358-T and rs7412-T, the APOE ε3 allele was identified by rs429358-T and rs7412-C, and the APOE ε4 allele was defined by rs429358-C and rs7412-C.

**Validation Analysis**

We evaluated whether our main results were influenced by several confounding factors (e.g., the gray matter atrophy, connectivity threshold, head motion and potentially artificial local correlations).

*Gray Matter Atrophy*.

Previous studies suggest that functional analysis results in AD/aMCI could potentially be influenced by gray matter atrophy . To explore the possible confounding effects of the brain atrophy, we performed a voxel-based morphometry analysis on structural MRI images and took the gray matter density (i.e., unmodulated images) as a covariate in the network centrality analyses. Briefly, individual gray matter density maps in the standard space were obtained using a unified segmentation algorithm as described previously. After spatially smoothing with a 10-mm FWHM Gaussian kernel, a voxel-wise two-way ANCOVA was conducted to examine between-group differences in brain morphology, with age, gender and years of education as covariates. Statistical significance was set at *P <* 0.05 and cluster size > 43,119 mm^3^, which corresponded to a corrected *P* < 0.05. Finally, we re-performed voxel-by-voxel ANCOVA analyses on individual DC and EC maps to examine the diagnosis-by-genotype interactions by adding individual gray matter density values as an additional covariate.

*Connectivity Thresholds.*

We used a single correlation coefficient threshold of 0.2 to eliminate weak correlations possibly arising from noise signals during the DC and EC analyses. This analysis led to different connectivity numbers across subjects. To determine whether our main results depended on the choice of connectivity threshold, we re-computed the DC and EC maps and performed corresponding statistical analyses under several network densities or sparsities (e.g., 5% and 10%), ensuring the same number of connections across all subjects.

*Head Motion*.

Several recent R-fMRI studies have reported influences of head motion on functional connectivity analysis [^3-6^](#_ENREF_3). In this study, we found that there were no significant effects of diagnosis and APOE genotype and the diagnosis-by-genotype interactions on the maximum translational and rotational movements and mean frame-wise displacement [^4^](#_ENREF_4) (all *Ps* > 0.05). Nonetheless, we conservatively evaluated the effects of head motion on our results using the “scrubbing” method . Briefly, we first calculated the frame-wise displacement between the neighboring volumes within each subject, and then scrubbed the volumes with a frame-wise displacement above 0.5 mm and their 2 forward and 1 back volumes for each subject. Then, the DC and EC analyses were re-performed using the resultant scrubbed R-fMRI data.

*Potentially Artificial Local Correlations*.

Local correlations between nearby voxels can arise from shared patterns of local neuronal activity, but they can also arise from aspects of data processing (e.g., reslicing, blurring) and motion-induced artifacts [^4^](#_ENREF_4). Local correlations are thus combinations of neurobiological and artifactual signals. To minimize the effects of questionable correlations on network structure, modified voxel-wise networks were presented in which very local correlations terminating within 10 mm of the source voxel were excluded. Then, we re-computed the DC and EC maps and performed the corresponding statistical analyses.

**Supplemental Results**

**
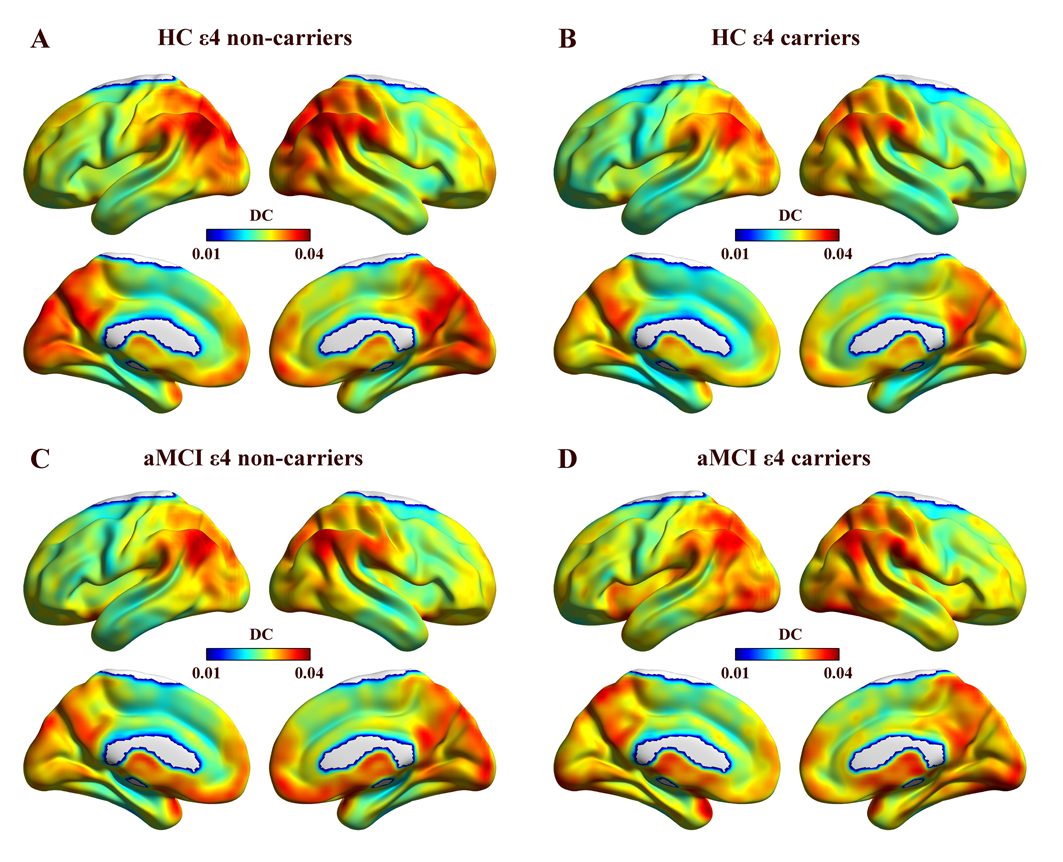
**

**Figure S1.** Spatial patterns of whole-brain DC maps in the four subgroups. **(A)** APOE ε4 non-carriers in the HC group. **(B)** APOE ε4 carriers in the HC group. **(C)** APOE ε4 non-carriers in the aMCI group. **(D)** APOE ε4 carriers in the aMCI group. The surface maps were made using the BrainNet Viewer (http://www.nitrc.org/projects/bnv). The color bar represents the strength of DC. DC, degree centrality; HC, healthy control; and aMCI, amnestic mild cognitive impairment.


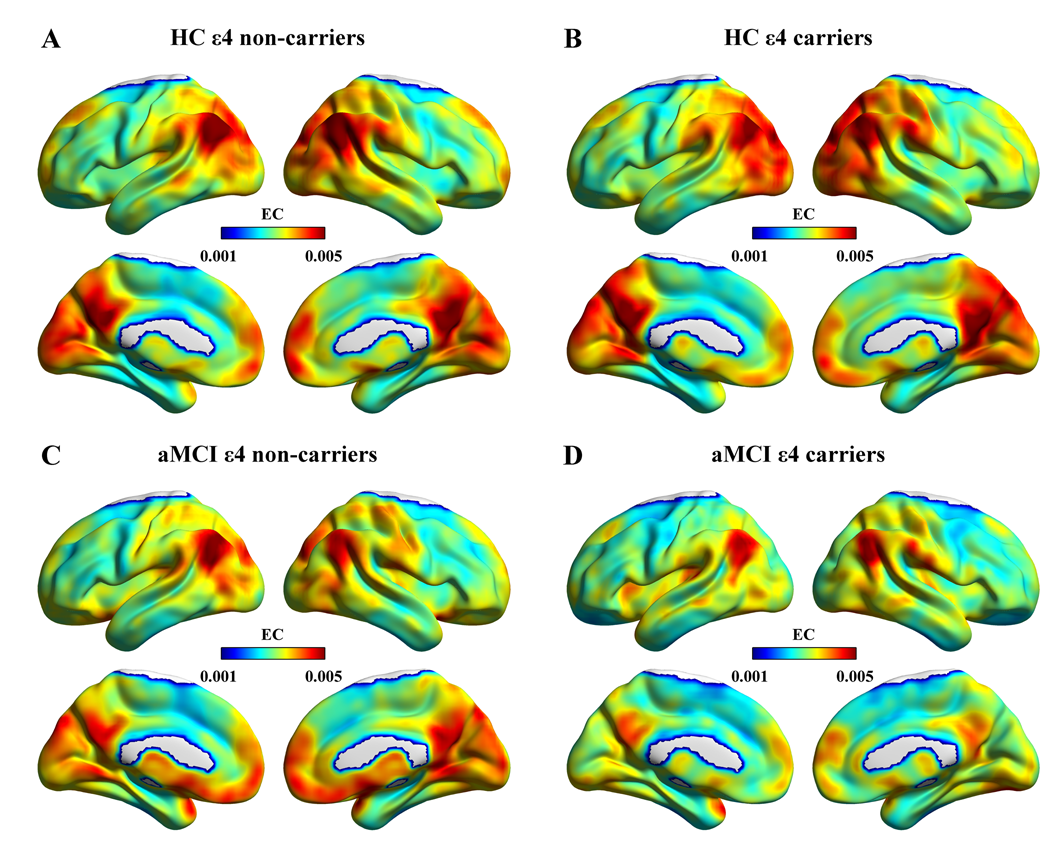


**Figure S2.** Spatial patterns of whole-brain EC maps in the four subgroups. **(A)** APOE ε4 non-carriers in the HC group. **(B)** APOE ε4 carriers in the HC group. **(C)** APOE ε4 non-carriers in the aMCI group. **(D)** APOE ε4 carriers in the aMCI group. The surface maps were made using the BrainNet Viewer (http://www.nitrc.org/projects/bnv). The color bar represents the strength of EC. EC, eigenvector centrality; HC, healthy control; and aMCI, amnestic mild cognitive impairment.

**Validation Results**

We evaluated whether our main results were influenced by several confounding factors (e.g., the gray matter atrophy, connectivity threshold, head motion and potentially artificial local correlations).

1. *The effects of gray matter atrophy*.

Significant effects of diagnosis but not APOE genotype were observed: compared with the HCs, the patients with aMCI showed significant gray matter loss in the bilateral hippocampal and parahippocampal gyrus (Figure S3 A and B). In this present study, we observed a reduction in the volume of medial temporal lobe structures (e.g., hippocampal and parahippocampal gyrus) in aMCI, supporting the idea that medial temporal lobe atrophy may serve as a potential imaging biomarker of AD-related brain changes. In addition, some [^7-9^](#_ENREF_7), but not all [^10^](#_ENREF_10), structural imaging studies also have shown reduced brain volume in the posterior cingulate cortex in patients with aMCI, suggesting that atrophy in posterior cingulate cortex is another potential biomarker for AD. The apparent lack of an aMCI-related GM atrophy in the posterior cingulate cortex in this present study may be due to the sample characteristics: the subjects for the present study were recruited from a community based cohort whereas many of previous studies were hospital based. More importantly, previous AD studies have suggested a well-described pattern of gray matter atrophy mainly affecting first the medial and then the lateral temporal areas, before extending to the cingulate cortegx and temporoparietal regions [^11-14^](#_ENREF_11). Longitudinal studies are needed to be conducted to examine the gray matter atrophy pattern in patients with aMCI.

We further observed significant diagnosis-by-genotype interactions on gray matter density in the right hippocampal/parahippocampal gyrus extending to temporal pole and bilateral anterior/middle cingulate gyrus (Figure S3 C). Post-hoc pairwise analysis revealed that the ε4 carriers showed significant gray matter loss in these regions compared with the non-carriers in the aMCI group, but not in the HC group. Notably, our main results in functional analyses were largely preserved when controlling for regional gray matter density (Figure S4).

1. *The effects of connectivity thresholds*.

We found that the diagnosis-by-genotype interactions on DC and EC maps under different thresholds were similar to our main results (Figure S5: A, sparsity = 5%; B, sparsity = 10%).

1. *The effects of head motion*.

To determine the extent to which our findings were robust to motion correction strategy, we repeated network centrality analyses using the scrubbed R-fMRI data and found that our main results identified in primary analyses were not affected (Figure S6). Note that in this scrubbing analysis, to have sufficient time points for stable results, subjects with ≤ 5 min of data remaining after censoring were excluded from the analysis (2 aMCI patients and 4 healthy controls were excluded by this criterion; 150 of 156 participants remained).

1. *The effects of potentially artificial local correlations*.

To explore the effects of potentially artificial local correlations on our results, we first presented modified voxel-wise networks in which all ties terminating within 10 mm of a source voxel were excluded. Based on the modified voxel-wise networks, we then repeated network analyses and found that our main results were largely preserved (Figure S7).


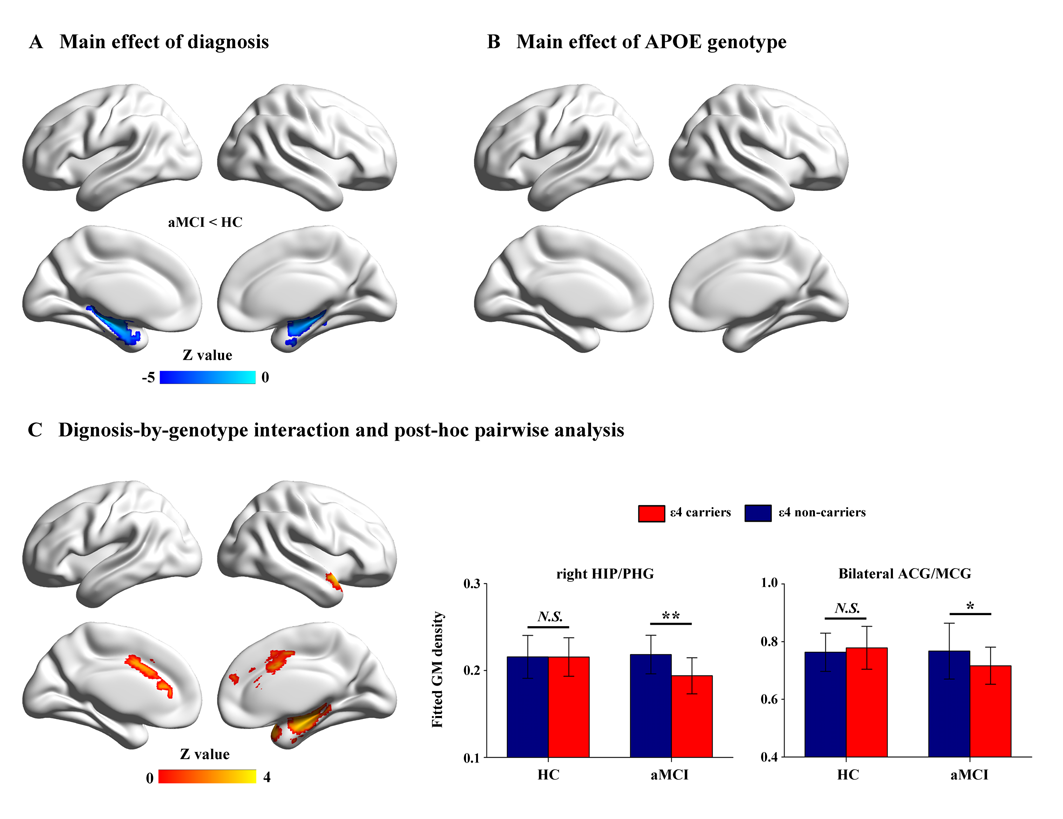


**Figure S3.** Statistical maps of voxel-based morphometry analysis. **(A)** Main effect of diagnosis on gray matter density. The color map shows significant differences in *Z* between the aMCI and HC groups after correction for multiple comparisons using the AlphaSim correction at *P* < 0.05. Cool colors represent decreased gray matter density in the aMCI group compared with the HC group. **(B)** Main effect of APOE genotype on gray matter density. There was no significant main effect of APOE genotype. **(C)** Diagnosis-by-genotype interactions on gray matter density. Significant interactions of diagnosis and APOE genotype were observed in the right hippocampal/parahippocampal gyrus (HIP/PHG) extending to temporal pole and bilateral anterior/middle cingulate gyrus (ACG/MCG). The color bar represents the statistical significance threshold (*Z*-score). The bar graphs describe the post-hoc pairwise comparisons from the diagnosis-by-genotype interactions. The differences in gray matter density between the ε4 carriers and non-carriers were significant in the aMCI group but not in the HC group. The data were expressed as the mean (M) ± standard deviation (SD). GM, gray matter; HC, healthy control; and aMCI, amnestic mild cognitive impairment. N.S., Non-significant. * *P* < 0.05, ** *P* < 0.01.


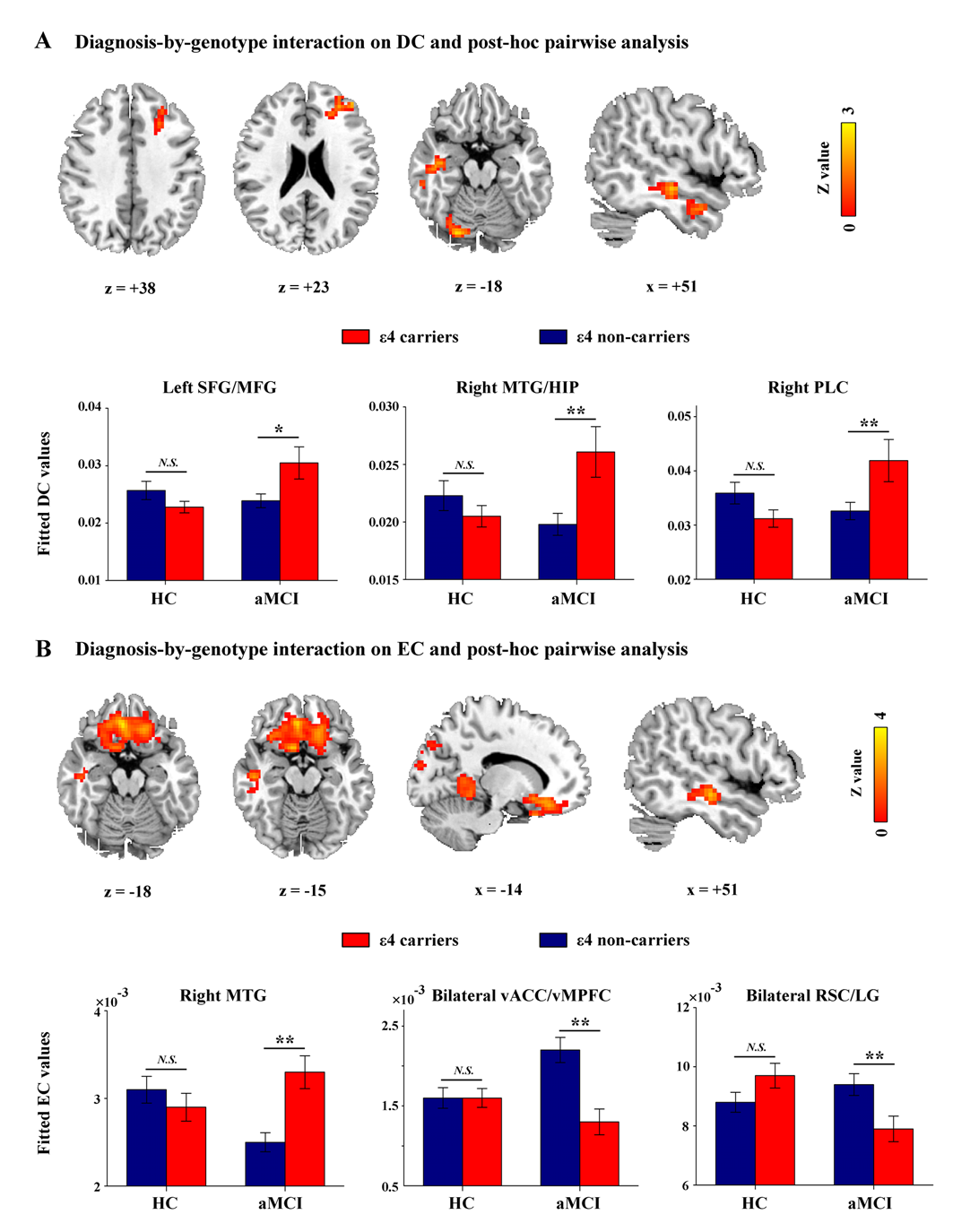


**Figure S4.** Validation results controlling for the effects of gray matter atrophy. The major results of the diagnosis-by-genotype interactions on DC **(A)** and EC **(B)** were reproducible after regressing out the regional gray matter density. Notably, the diagnosis-by-genotype interactions on DC in the right PLC and EC in the right MTG survived the height but not the extent threshold (3,483 mm^3^; 3,564 mm^3^). DC, degree centrality; EC, eigenvector centrality; HC, healthy control; aMCI, amnestic mild cognitive impairment; SFG/MFG, superior/middle frontal gyrus; MTG, middle temporal gyrus; PLC, posterior lobe of the cerebellum; vACC/vMPFC, ventral anterior cingulate/ventral medial prefrontal cortex; and RSC, retrosplenial cortex. N.S., Non-significant. * *P* < 0.05, ** *P* < 0.01.

**
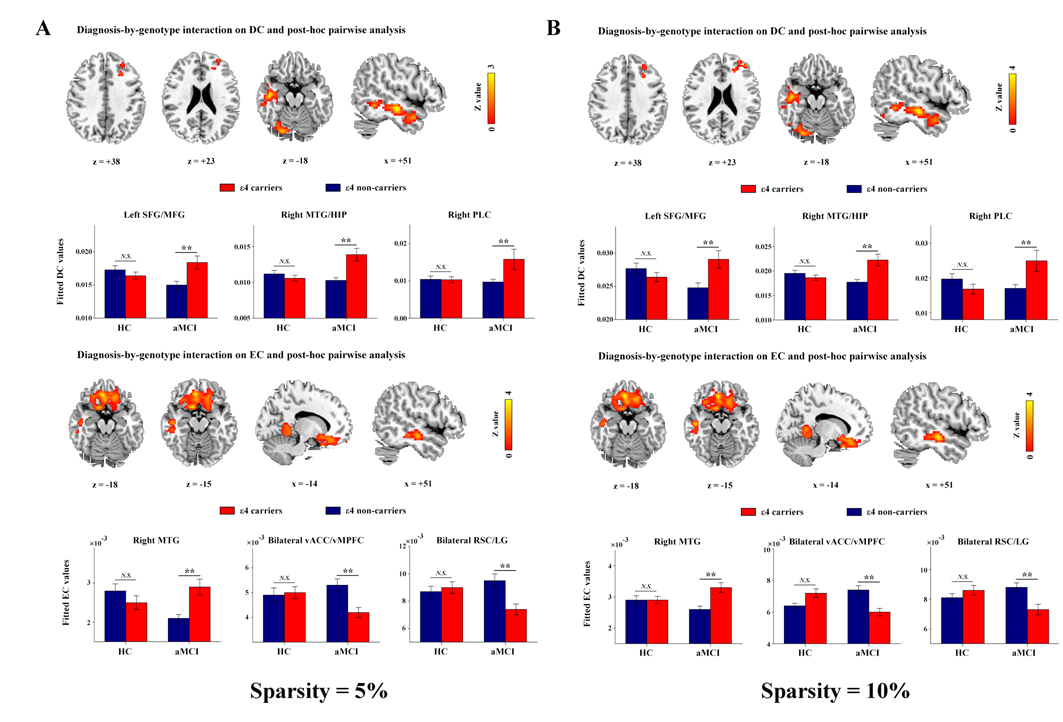
**

**Figure S5.** Validation results under different network sparsities. This figure shows the diagnosis-by-genotype interactions on DC and EC when using different network sparsities. **(A)** The threshold was set at sparsity = 5%. **(B)** The threshold was set at sparsity = 10%. DC, degree centrality; EC, eigenvector centrality; HC, healthy control; aMCI, amnestic mild cognitive impairment; SFG/MFG, superior/middle frontal gyrus; MTG, middle temporal gyrus; PLC, posterior lobe of cerebellum; vACC/vMPFC, ventral anterior cingulate/ventral medial prefrontal cortex; and RSC, retrosplenial cortex. N.S., Non-significant. ** *P* < 0.01.


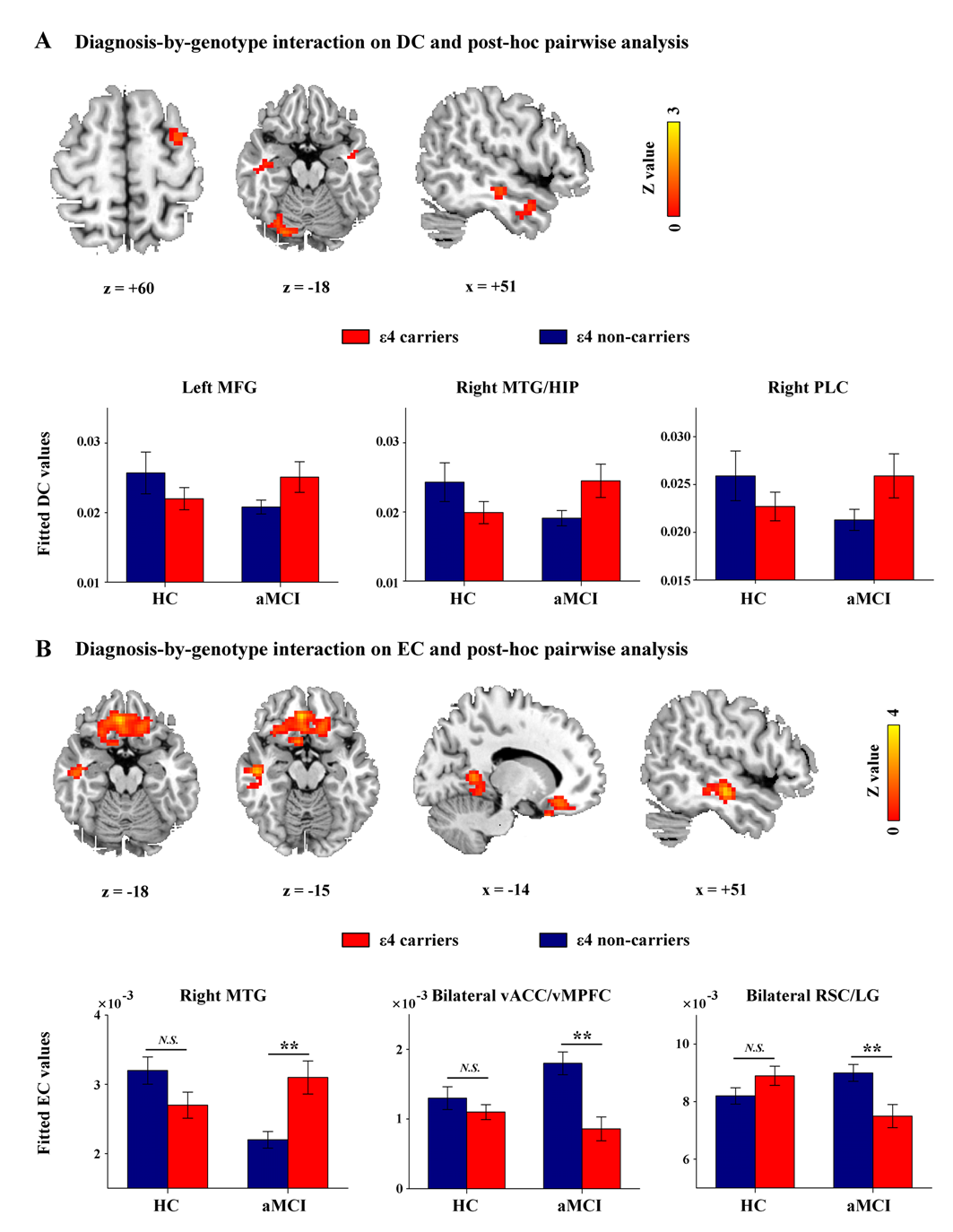


**Figure S6.** Validation results with the “scrubbing” procedure for head motion correction in the preprocessing steps. The major results of the diagnosis-by-genotype interactions on DC **(A)** and EC **(B)** were reproducible with head-motion scrubbing. Notably, the diagnosis-by-genotype interactions on DC were observed in the left MFG, right MTG and PLC at an uncorrected threshold of *P* < 0.10. Additionally, the diagnosis-by-genotype interactions on EC in the right MTG survived the height but not the extent threshold (3,807 mm^3^). DC, degree centrality; EC, eigenvector centrality; HC, healthy control; aMCI, amnestic mild cognitive impairment; SFG/MFG, superior/middle frontal gyrus; MTG, middle temporal gyrus; PLC, posterior lobe of the cerebellum; vACC/vMPFC, ventral anterior cingulate/ventral medial prefrontal cortex; and RSC, retrosplenial cortex. N.S., Non-significant. ** *P* < 0.01.


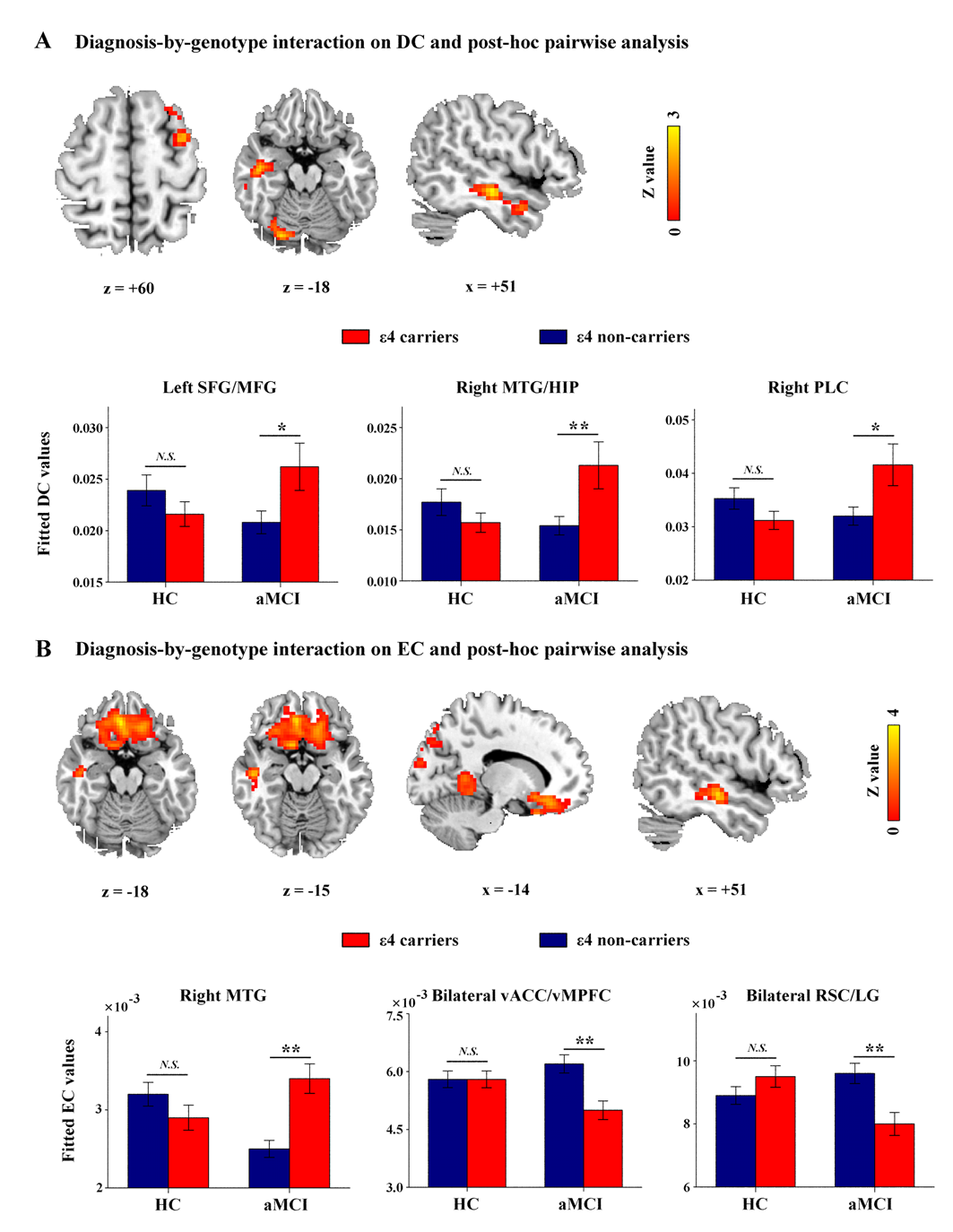


**Figure S7.** Validation results with removal of the artificial local correlations when constructing the voxel-wise networks. The major results of the diagnosis-by-genotype interactions on DC **(A)** and EC **(B)** were reproducible while controlling for the effects of artificial local correlations between nearby voxels. Notably, the diagnosis-by-genotype interactions on DC in the left SFG/MFG and EC in the right MTG survived the height but not the extent threshold (1,944 mm^3^; 3,699 mm^3^). DC, degree centrality; EC, eigenvector centrality; HC, healthy control; aMCI, amnestic mild cognitive impairment; SFG/MFG, superior/middle frontal gyrus; MTG, middle temporal gyrus; PLC, posterior lobe of the cerebellum; vACC/vMPFC, ventral anterior cingulate/ventral medial prefrontal cortex; and RSC, retrosplenial cortex. N.S., Non-significant. * *P* < 0.05, ** *P* < 0.01.

**References:**

1 Wang, Z. *et al.* Spatial patterns of intrinsic brain activity in mild cognitive impairment and Alzheimer's disease: a resting-state functional MRI study. *Hum Brain Mapp* **32**, 1720-1740 (2011).

2 He, Y. *et al.* Regional coherence changes in the early stages of Alzheimer's disease: a combined structural and resting-state functional MRI study. *Neuroimage* **35**, 488-500 (2007).

3 Satterthwaite, T. D. *et al.* An improved framework for confound regression and filtering for control of motion artifact in the preprocessing of resting-state functional connectivity data. *Neuroimage* **64**, 240-256 (2013).

4 Power, J. D. *et al.* Spurious but systematic correlations in functional connectivity MRI networks arise from subject motion. *Neuroimage* **59**, 2142-2154 (2012).

5 Van Dijk, K. R., Sabuncu, M. R. & Buckner, R. L. The influence of head motion on intrinsic functional connectivity MRI. *Neuroimage* **59**, 431-438 (2012).

6 Yan, C. G. *et al.* A comprehensive assessment of regional variation in the impact of head micromovements on functional connectomics. *Neuroimage* **76**, 183-201 (2013).

7 Choo, I. H. *et al.* Posterior cingulate cortex atrophy and regional cingulum disruption in mild cognitive impairment and Alzheimer's disease. *Neurobiol Aging* **31**, 772-779 (2010).

8 Pengas, G., Hodges, J. R., Watson, P. & Nestor, P. J. Focal posterior cingulate atrophy in incipient Alzheimer's disease. *Neurobiol Aging* **31**, 25-33 (2010).

9 Bailly, M. *et al.* Precuneus and Cingulate Cortex Atrophy and Hypometabolism in Patients with Alzheimer's Disease and Mild Cognitive Impairment: MRI and (18)F-FDG PET Quantitative Analysis Using FreeSurfer. *Biomed Res Int* **2015**, 583931 (2015).

10 Gili, T. *et al.* Regional brain atrophy and functional disconnection across Alzheimer's disease evolution. *J Neurol Neurosurg Psychiatry* **82**, 58-66 (2011).

11 Matsuda, H. *et al.* Longitudinal evaluation of both morphologic and functional changes in the same individuals with Alzheimer's disease. *J Nucl Med* **43**, 304-311 (2002).

12 Chetelat, G. *et al.* Dissociating atrophy and hypometabolism impact on episodic memory in mild cognitive impairment. *Brain* **126**, 1955-1967 (2003).

13 Thompson, P. M. *et al.* Dynamics of gray matter loss in Alzheimer's disease. *J Neurosci* **23**, 994-1005 (2003).

14 Whitwell, J. L. *et al.* 3D maps from multiple MRI illustrate changing atrophy patterns as subjects progress from mild cognitive impairment to Alzheimer's disease. *Brain* **130**, 1777-1786 (2007).
